# The Effect of Liver Cirrhosis on Patients Undergoing Cardiac Surgery

**DOI:** 10.5334/gh.1270

**Published:** 2023-10-05

**Authors:** Fei Liu, Zi-Wei Li, Xu-Rui Liu, Xiao-Yu Liu, Jun Yang

**Affiliations:** 1 Department of Gastrointestinal Surgery, the First Affiliated Hospital of Chongqing Medical University, Chongqing, China; 2 Department of Anesthesiology, The First Affiliated Hospital of Chongqing Medical University, Chongqing, China

**Keywords:** liver cirrhosis, cardiac surgery, complications

## Abstract

The aim of this study was to investigate the impact of liver cirrhosis (LC) on postoperative complications and long-term outcomes in patients who underwent cardiac surgery. Three databases, including PubMed, Embase, and the Cochrane Library, were searched on July 24, 2022. A total of 1,535,129 patients were enrolled in the seven included studies for analysis. According to our analysis, LC was a risk factor for postoperative overall complications (OR = 1.48, 95% CI = 1.21 to 1.81, I^2^ = 90.35%, P = 0.00 < 0.1). For various complications, more patients developed pulmonary (OR = 1.86, 95% CI = 1.21 to 2.87, I^2^ = 90.79%, P = 0.00 < 0.1), gastrointestinal (OR = 2.03, 95% CI = 1.32 to 3.11, I^2^ = 0.00%, P = 0.00 < 0.05), renal (OR = 2.20, 95% CI = 1.41 to 3.45, I^2^ = 91.60%, P = 0.00 < 0.1), neurological (OR = 1.14, 95% CI = 1.03 to 1.26, I^2^ = 7.35%, P = 0.01 < 0.05), and infectious (OR = 2.02, 95% CI = 1.17 to 3.50, I^2^ = 92.37%, P = 0.01 < 0.1) complications after surgery in the LC group. As for cardiovascular (OR = 1.07, 95% CI = 0.85 to 1.35, I^2^ = 75.23%, P = 0.58 > 0.1) complications, there was no statistical significance between the 2 groups. As for long-term outcomes, we found that in-hospital death (OR = 2.53, 95% CI = 1.86 to 3.20, I^2^ = 44.58%, P = 0.00 < 0.05) and death (OR = 3.31, 95% CI = 1.54 to 5.07, I^2^ = 93.81%, P = 0.00 < 0.1) in the LC group were higher than the non-LC group. LC was a risk factor for cardiac surgery. Patients with LC who would undergo cardiac surgery should be fully assessed for the risks of cardiac surgery. Similarly, the surgeon should assess the patient’s liver function before surgery.

## Background

Liver cirrhosis (LC) is a common disease that kills about 1.03 million people each year [1,2]. Patients with LC often suffered from nutritional damage, immune system dysfunction, coagulation disorders, acute kidney injury, etc. [3,4,5]. These issues cause surgeons to be hesitant to operate on patients with LC. Surgery on patients with LC remains a challenge for surgeons and anaesthetists. This challenge depends on the type of liver disease and its severity, the surgical procedure, and the type of anaesthesia [6,7].

As for cardiothoracic surgery, especially cardiac surgery requiring cardiopulmonary bypass, LC remains a tricky problem [8,9,10]. Liver disease remains a major risk factor in the perioperative period of cardiac surgery [11]. Cardiac disease could be a fatal condition. Surgery was an excellent treatment. As for LC patients, the surgeon should assess the patient’s liver function before surgery. The underlying physiological conditions caused by LC make these patients vulnerable to coagulation dysfunction and major organ dysfunction after direct cardiac surgery with extracorporeal circulation [12].

Figuring out the impact and mechanism of LC on cardiac surgery could help surgeons find preventive measures. However, according to our review of previous studies, the effects of LC on the postoperative outcomes of cardiac surgery continued to be controversial. Some studies suggest a poor effect of LC on postoperative outcomes in cardiac surgery [13,14,15,16,17]. Other studies demonstrated that there was no association between LC and postoperative cardiac surgery outcomes [18,19]. Therefore, this pooling-up analysis aimed to investigate the impact of LC on postoperative complications and long-term outcomes in patients who underwent cardiac surgery.

## Methods

Our meta-analysis was produced in accordance with the Preferred Reporting Items for Systematic Reviews and Meta-Analyses (PRISMA) statement [20]. Three databases were searched, including PubMed, Embase, and the Cochrane Library, on July 24, 2022. The key words of the search strategy were LC and cardiac surgery. The search strategy for LC was as follows: “liver cirrhosis” OR “cirrhosis” OR “cirrhotic”, and as for cardiac surgery, we searched “cardiac surgery” OR “cardiac operation” OR “heart surgery” OR “heart operation” OR “thoracic surgery” OR “cardiac surgical procedures” OR “cardiopulmonary bypass” OR “CPB” OR “congenital heart disease”. Then, the two search strategies were combined by “AND”. The search was limited to titles and abstracts, and the language was limited to English.

The inclusion criteria for eligible studies were as follows: 1) all patients were diagnosed with cardiac disease and underwent cardiac surgery; 2) both the LC group and the non-LC group were reported; 3) at least 1 of the following complications (cardiovascular, pulmonary, gastrointestinal, renal, neurological, infectious) was reported; and 4) as for long-term outcomes, in-hospital death or death should be reported. The exclusion criteria were as follows: 1) case reports, case series, comments, letters to the editor, conference abstracts, and nonoriginal articles; 2) data was repeated or overlapped; and 3) incomplete information. Two authors searched the databases and identified eligible studies separately. First, duplicate studies were excluded. Then, the two authors scanned the titles and abstracts to find eligible studies. Finally, the full text would be read to identify studies that could be included. Any disagreements were settled by a third author.

Patients were divided into the LC group and the non-LC group according to whether they were diagnosed with LC. The cardiac surgery types included coronary artery bypass graft, surgery with cardiopulmonary bypass, cardiac surgery, and aortic valve replacement. The complication was defined as a cardiovascular, pulmonary, gastrointestinal, renal, neurological, or infectious disease that occurred after surgery. Overall complication was the sum of all complications reported in the included studies that were not directly reported. In-hospital death was defined as a patient’s cause of death when they died in the hospital after surgery. Death was defined as a patient’s cause of death after they left the hospital.

The information included characteristics of the studies, baseline information on patients, medical history, postoperative complications, and long-term outcomes. The characteristics of the studies were as follows: the first author, published year, published country, study date, sample size of the LC group and the non-LC group, and Newcastle-Ottawa Scale (NOS) score. The baseline information for patients included age, gender, and race. For medical history, hypertension, diabetes, chronic obstructive pulmonary disease (COPD), heart failure, prior myocardial infarction (MI), and malignancy were collected. For complications, cardiovascular, pulmonary, gastrointestinal, renal, neurological, and infectious diseases were collected. As for long-term outcomes, in-hospital death and death were collected.

In-hospital death was defined as a death that occurred during hospitalization, regardless of cause. Death was defined as a death that occurred after discharge from the hospital due to cardiac failure or liver cirrhosis. If the cause of death during this period was unknown, it was also considered related.

The NOS was used to assess the quality of the included studies [21]. High-quality studies would be scored at nine points. Median quality had scores ranging from 7 to 8 points. Low-quality studies were indicated by a score of less than 7 points.

The mean difference (MD) and 95% confidence interval (CI) were calculated for age. The odds ratio (OR) and 95% CI were calculated for gender, medical history, complications, and long-term outcomes. I^2^ values were used to assess the statistical heterogeneity of the included studies [22,23]. When I^2^ > 50%, which was considered to be high heterogeneity, the random effects model was used, and P < 0.1 was considered statistically significant. Otherwise, the fixed effects model would be used, and p < 0.05 meant statistically significant. We performed data analysis with Stata V16.0 software.

## Results

**Figure 1:**
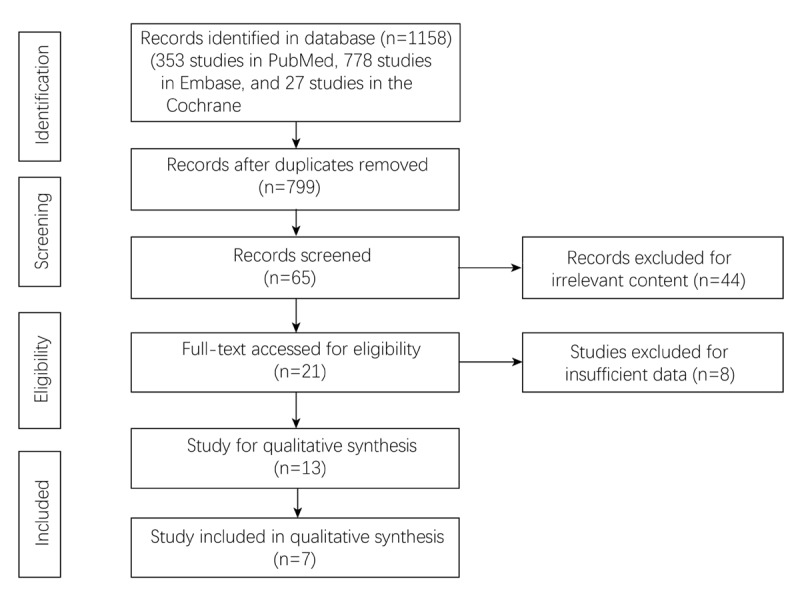
Flowchart of study selection.

A total of 1,158 studies were searched from the three databases (353 studies in PubMed, 778 studies in Embase, and 27 studies in the Cochrane Library). 359 duplicate studies were eliminated. After the remaining 799 studies were viewed for titles and abstracts, 21 studies were left for full-text screening. Then, there were 13 studies left for qualitative synthesis. Finally, seven eligible studies were included in this analysis [13,14,15,16,17,18,19] (Figure 1).

A total of 1,535,129 patients were included in this analysis using the seven included studies. All patients were divided into the LC group and the non-LC group (8,370 in the LC group and 1,526,759 in the non-LC group). The studies were published from 2009 to 2019. The study period was from 1984 to 2014. More information (authors, published countries, surgery type, sample size, and NOS score) is shown in Table 1.

**Table 1:** Characteristics of the studies included in the meta-analysis.


AUTHOR	YEAR	COUNTRY	STUDY DATE	SURGERY TYPE	SAMPLE SIZE		FOLLOW-UP (MONTHS)	NOS

					LC	NON-LC		

Shaheen AAM	2009	Canada	1998–2004	CABG	711	402383	156	9

MACARON C	2012	Florida	1992–2009	surgery with cardiopulmonary bypass	54	216	3	7

Ruiz-Morales J	2015	Spain	1984–2008	cardiac surgery	308	2828	360	8

Steffen RJ	2017	USA	1998–2011	AVR	2769	421020	156	9

Chou AH	2017	China	1997–2011	CABG	1040	1040	32	8

Singh V	2018	USA	1998–2004	CABG	2231	696568	156	9

Xavier S	2019	Canada	2004–2014	cardiac surgery	60	310	120	8


Abbreviations: LC, liver cirrhosis; CABG, coronary artery bypass graft; AVR, aortic valve replacement; NOS, Newcastle-Ottawa Scale.

After pooling up all the baseline information (including age, gender, and race), the outcomes showed a statistical difference in race (OR = 0.57, 95% CI = 0.50 to 0.65, I^2^ = 69.69%, P = 0.00 < 0.1) between the 2 groups. However, there were no statistical differences in age (MD = –8.47, 95% CI = –22.71 to 5.78, I^2^ = 100.00%, P = 0.24 > 0.1) or gender (OR = 1.10, 95% CI = 0.94 to 1.28, I^2^ = 64.11%, P = 0.25 > 0.1) (Table 2).

**Table 2:** Summary of characteristics between LC group and Non-LC group.


CHARACTERISTICS	STUDIES	PARTICIPANTS (LC/NON-LC)	MEAN DIFFERENCE/ODDS RATIO (95% CI)	MODEL	HETEROGENEITY

Baseline information					

Age, year	4	4580/824753	–8.47 [–22.71, 5.78]; P = 0.24	RE	I^2^ = 100.00%; P = 0.00

Gender, male	6	4404/1103345	1.10 [0.94, 1.28]; P = 0.25	RE	I^2^ = 64.11%; P = 0.02

Race, white	4	5765/1520187	0.57 [0.50, 0.65]; P = 0.00	RE	I^2^ = 69.69%; P = 0.02

Medical history					

Hypertension	4	6100/1118938	0.70 [0.47, 1.06]; P = 0.09	RE	I^2^ = 93.46%; P = 0.00

Diabetes	4	6100/1118938	1.37 [1.09, 1.71]; P = 0.01	RE	I^2^ = 90.59%; P = 0.00

COPD	3	6040/1118628	1.44 [1.22, 1.70]; P = 0.00	RE	I^2^ = 82.67%; P = 0.00

Heart failure	3	3869/422370	1.61 [0.63, 4.13]; P = 0.32	RE	I^2^ = 97.59%; P = 0.00

Prior MI	2	1100/1350	0.94 [0.75, 1.18]; P = 0.60	FE	I^2^ = 0.00%; P = 0.50

Malignancy	3	6040/1118628	1.22 [0.78, 1.91]; P = 0.37	RE	I^2^ = 88.47%; P = 0.00


Abbreviations: LC, liver cirrhosis; COPD, chronic obstructive pulmonary disease; MI, myocardial infarction; CI, confidence interval; RE, random-effects; FE, fixed-effects.

The medical history included hypertension, diabetes, COPD, heart failure, prior MI, and malignancy. After pooling up the data, there were a higher proportion of diabetes (OR = 1.37, 95% CI = 1.09 to 1.71, I^2^ = 90.59%, P = 0.01 < 0.1), COPD (OR = 1.44, 95% CI = 1.22 to 1.70, I^2^ = 82.67%, P = 0.00 < 0.1) and a lower proportion of hypertension (OR = 0.70, 95% CI = 0.47 to 1.06, I^2^ = 93.46%, P = 0.09 < 0.1) in the LC group. As for other medical history, heart failure (OR = 1.61, 95% CI = 0.63 to 4.13, I^2^ = 97.59%, P = 0.32 > 0.1), prior MI (OR = 0.94, 95% CI = 0.75 to 1.18, I^2^ = 0.00%, P = 0.60 > 0.05) and malignancy (OR = 1.22, 95% CI = 0.78 to 1.91, I^2^ = 88.47%, P = 0.37 > 0.1) had no statistical differences between the two groups (Table 2).

**Figure 2:**
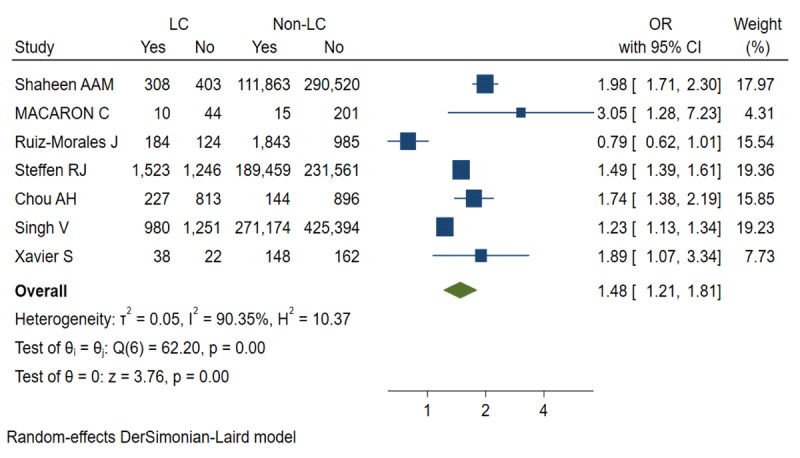
Overall complications of the LC group and the non-LC group. *Note*: LC, liver cirrhosis.

**Figure 3:**
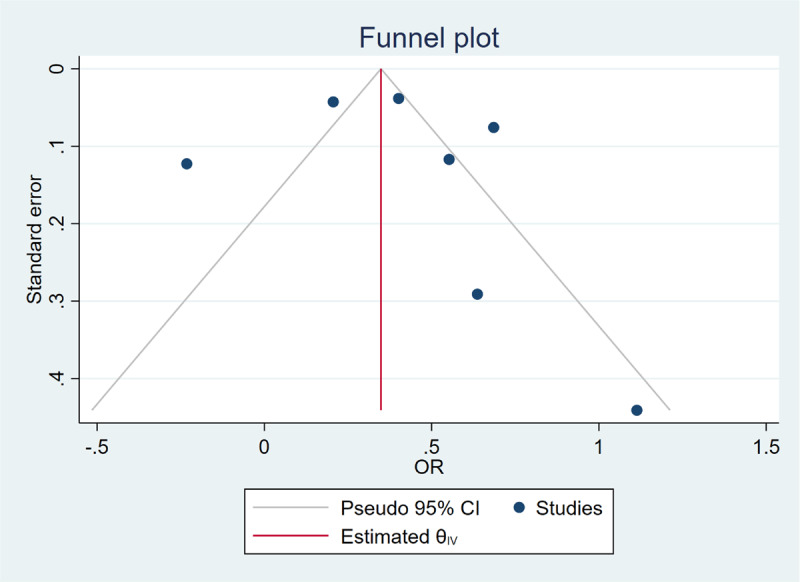
Funnel plot of overall complications.

The complications included cardiovascular, pulmonary, gastrointestinal, renal, neurological, and infectious diseases. According to our analysis, LC was a risk factor for postoperative overall complications (OR = 1.48, 95% CI = 1.21 to 1.81, I^2^ = 90.35%, P = 0.00 < 0.1) (Figure 2). Figure 3 shows the publication bias of the studies included. For various complications, more patients developed pulmonary (OR = 1.86, 95% CI = 1.21 to 2.87, I^2^ = 90.79%, P = 0.00 < 0.1), gastrointestinal (OR = 2.03, 95% CI = 1.32 to 3.11, I^2^ = 0.00%, P = 0.00 < 0.05), renal (OR = 2.20, 95% CI = 1.41 to 3.45, I^2^ = 91.60%, P = 0.00 < 0.1), neurological (OR = 1.14, 95% CI = 1.03 to 1.26, I^2^ = 7.35% P = 0.01 < 0.05), and infectious (OR = 2.02, 95% CI = 1.17 to 3.50, I^2^ = 92.37%, P = 0.01 < 0.1) complications after surgery in the LC group. As for cardiovascular (OR = 1.07, 95% CI = 0.85 to 1.35, I^2^ = 75.23%, P = 0.58 > 0.1) complication, no statistical significance was found between the 2 groups (Table 3).

**Table 3:** Summary of outcomes between LC group and Non-LC group.


CHARACTERISTICS	STUDIES	PARTICIPANTS (LC/NON-LC)	HAZARD RATIO/ODDS RATIO (95% CI)	MODEL	HETEROGENEITY

Complications					

Any	7	7173/1524365	1.48 [1.21, 1.81]; P = 0.00	RE	I^2^ = 90.35%; P = 0.00

Cardiovascular	4	3310/1102089	1.07 [0.85, 1.35]; P = 0.58	RE	I^2^ = 75.23%; P = 0.01

Pulmonary	3	3002/1099261	1.86 [1.21, 2.87]; P = 0.00	RE	I^2^ = 90.79%; P = 0.00

Gastrointestinal	2	771/402693	2.03 [1.32, 3.11]; P = 0.00	FE	I^2^ = 0.00%; P = 0.59

Renal	5	3364/1102305	2.20 [1.41, 3.45]; P = 0.00	RE	I^2^ = 91.60%; P = 0.00

Neurological	3	2599/699706	1.14 [1.03, 1.26]; P = 0.01	FE	I^2^ = 7.35%; P = 0.34

Infectious	4	4042/1100301	2.02 [1.17, 3.50]; P = 0.01	RE	I^2^ = 92.37%; P = 0.00

Long-term outcomes					

In-hospital death	4	NA	2.53 [1.86, 3.20]; P = 0.00	FE	I^2^ = 44.58%; P = 0.16

Death	4	NA	3.31 [1.54, 5.07]; P = 0.00	RE	I^2^ = 93.81%; P = 0.00


Abbreviations: LC, liver cirrhosis; ICU, intensive care unit; OS, overall survival; CI, confidence interval; RE, random-effects; FE, fixed-effects; NA, not apply.

According to our data analysis, we found that in-hospital death (OR = 2.53, 95% CI = 1.86 to 3.20, I^2^ = 44.58%, P = 0.00 < 0.05) and death (OR = 3.31, 95% CI = 1.54 to 5.07, I^2^ = 93.81%, P = 0.00 < 0.1) in the LC group were higher than the non-LC group (Table 3).

**Figure 4:**
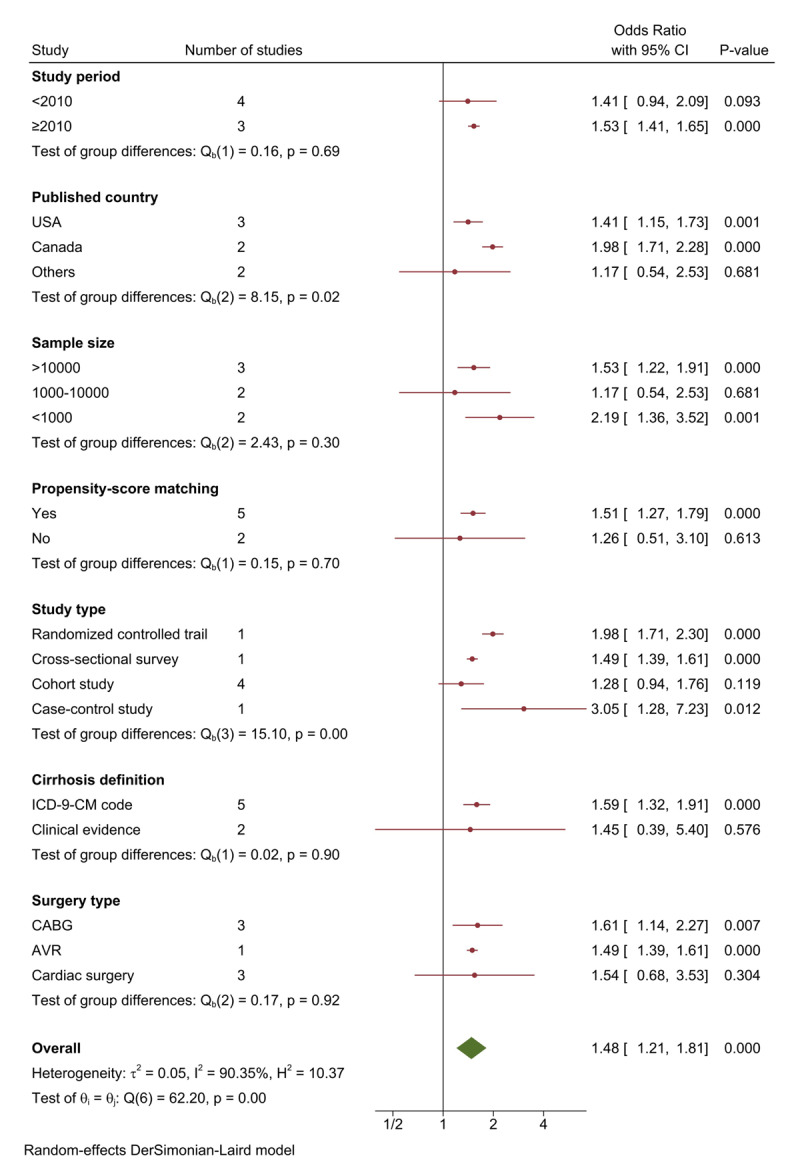
Meta regression analysis of covariates.

Meta subgroup analysis was conducted to find out why there was a high heterogeneity among the included studies. The covariates included study period, published country, sample size, propensity-score matching, study type, cirrhosis definition, and surgery type. After performing data analysis, we found that heterogeneity derived from the published country and the study type (OR = 1.48, 95% CI = 1.21 to 1.81, I^2^ = 90.35%, P = 0.00 < 0.1) (Figure 4).

Meta-analysis was repeated to analyze the sensitivity by excluding each study at a time. The results were not significantly different after every analysis.

## Discussion

The aim of this pooling-up analysis was to figure out if there was an impact of LC on cardiac surgery. A total of seven studies containing 1,535,129 patients who underwent cardiac surgery were included [13,14,15,16,17,18,19]. All seven studies reported LC and non-LC groups. After analysis, we found that LC was a risk factor for cardiac surgery. In terms of overall complications, the LC group was higher than the non-LC group. However, we also found that both in-hospital death and death in the LC group were higher.

LC was divided into three classes (Child-Pugh (CP) A, CP-B, and CP-C) according to the impairment of liver function. As previous studies suggested, mortality rates after cardiac surgery in patients with CP-A, CP-B, and CP-C cirrhosis were 0%–11%, 18%–50%, and 67%–100%, respectively [24,25,26,27]. One review of 19 studies reported that the mortality rates in patients with CP-A, CP-B, and CP-C cirrhosis were 9.6%, 33.9%, and 61.3% after cardiac surgery, respectively. Moreover, the total mortality rate was 20% after cardiac surgery in patients with LC [11]. In this pooling-up analysis, more patients developed pulmonary, gastrointestinal, renal, or infectious complications after surgery in the LC group. As for cardiovascular and neurological complications, there was no statistical significance between the two groups. One study suggested that the CP-A class was not associated with a poor prognosis [18]. Perhaps positive liver function was a condition for patient recovery.

Controversy remained over the relationship between LC and cardiac surgery. Among the included studies, some reported that patients with LC had a poor prognosis after cardiac surgery [13,14,15,16,17]. One of these studies divided the LC patients into two groups, which were bound by the CP score [19]. Another study discussed the impact of LC on cardiac surgery by dividing it into CP-A, CP-B, and CP-C groups [18]. Both concluded that the group with positive liver function (CP-A or CP-B, CP score less than eight) could safely perform cardiac surgery, while the group with poor liver function (CP-B or CP-C, CP score more than or equal to eight) was responsible for the poor prognosis of cardiac surgery. Based on this controversy, we carried out this pooling up-analysis.

The potential mechanism was that patients with LC often suffered from nutritional damage, immune system dysfunction, coagulation disorders, acute kidney injury, etc. [1,2,3]. These concomitant symptoms might be the cause of a poor prognosis for the patients. Meanwhile, many confounding factors exist, including but not limited to the type of surgery, the emergency nature of the surgery, potential co-morbidities, and the year of the surgery, as surgical techniques have changed over the past decade [28,29,30]. The impact of non-LC factors on cardiac surgery can also be significant. Therefore, additional risk factors needed to be discussed and analyzed.

To our knowledge, this study was the first to utilize pooling-up analysis. However, there were some limitations to this analysis. First, we did not discuss in depth the impact of individual liver function classes (CP-A, CP-B, CP-C, or other classification methods) on cardiac surgery. Therefore, the data on CP-A was insufficient. Second, the heterogeneity of the included studies was high. After subgroup analysis, we found that the sources of heterogeneity were the publication of nation and study type. Although there was a high heterogeneity among the included studies, the sensitivity analysis of these studies did not affect our results. Third, we lacked information about the severity of liver disease, which could strengthen the analysis significantly. Fourth, the earliest and most recent publication dates for the included studies were 2009 and 2020, respectively. In modern conditions, the results of operations could differ due to the improvement of anesthetic and surgical techniques. However, there were no relevant studies in the last three years.

In conclusion, LC was a risk factor of cardiac surgery. More attention should be paid to LC patients after cardiac surgery. Similarly, the surgeon should assess the patient’s liver function before surgery.

## Data Availability Statement

The data was accessed in the database.

## Abbreviations

LC, liver cirrhosis; OR, odds ratio; MD, mean difference; CI, confidence interval; NOS, Newcastle-Ottawa Scale; COPD, chronic obstructive pulmonary disease; MI, myocardial infarction; CP, Child-Pugh.

## References

[1] Cheng YX, Tao W, Zhang H, Peng D, Wei ZQ. Does liver cirrhosis affect the surgical outcome of primary colorectal cancer surgery? A meta-analysis. World J Surg Oncol. 2021; 19(1): 167. DOI: 10.1186/s12957-021-02267-6 34107967 PMC8191032

[2] Liu XR, Li LS, Liu F, Li ZW, Liu XY, Zhang W, et al. Short-term and long-term outcomes of liver cirrhosis in gastric neoplasm patients undergoing endoscopic submucosal dissection. J Laparoendosc Adv Surg Tech A. 2023; 10: 1089. DOI: 10.1089/lap.2023.0022 36946655

[3] Lopez-Delgado JC, Esteve F, Javierre C, Ventura JL, Mañez R, Farrero E, et al. Influence of cirrhosis in cardiac surgery outcomes. World J Hepatol. 2015; 7(5): 753–760. DOI: 10.4254/wjh.v7.i5.753 25914775 PMC4404380

[4] Wannhoff A, Hippchen T, Weiss CS, Friedrich K, Rupp C, Neumann-Haefelin C, et al. Cardiac volume overload and pulmonary hypertension in long-term follow-up of patients with a transjugular intrahepatic portosystemic shunt. Aliment Pharmacol Ther. 2016; 43(9): 955–965. DOI: 10.1111/apt.13569 26919285

[5] Dourakis SP, Geladari E, Geladari C, Vallianou N. Cirrhotic cardiomyopathy: The interplay between liver and cardiac muscle. How does the cardiovascular system react when the liver is diseased? Curr Cardiol Rev. 2021; 17(1): 78–84. DOI: 10.2174/1573403X15666190509084519 31072296 PMC8142364

[6] Sabbagh C, Fuks D, Regimbeau JM. Non-hepatic gastrointestinal surgery in patients with cirrhosis. J Visc Surg. 2014 Jun; 151(3): 203–11. DOI: 10.1016/j.jviscsurg.2014.04.004 24810712

[7] Hoetzel A, Ryan H, Schmidt R. Anesthetic considerations for the patient with liver disease. Curr Opin Anaesthesiol. 2012 Jun; 25(3): 340–7. DOI: 10.1097/ACO.0b013e3283532b02 22450699

[8] Wallwork K, Ali JM, Abu-Omar Y, De Silva R. Does liver cirrhosis lead to inferior outcomes following cardiac surgery? Interact Cardiovasc Thorac Surg. 2019 Jan 1; 28(1): 102–107. DOI: 10.1093/icvts/ivy221 30052992

[9] Garatti A, Daprati A, Cottini M, Russo CF, Dalla Tomba M, Troise G, et al; Italian Group of Research for Outcome in Cardiac Surgery (GIROC). Cardiac Surgery in Patients With Liver Cirrhosis (CASTER) Study: Early and long-term outcomes. Ann Thorac Surg. 2021 Apr; 111(4): 1242–1251. DOI: 10.1016/j.athoracsur.2020.06.110 32919974

[10] Garatti A. Italian Group of Research for Outcome in Cardiac Surgery (GIROC). Cardiac Surgery in Patients With Liver Cirrhosis: Can we paint all patients with the same brush? Ann Thorac Surg. 2022 Aug; 114(2): 605–606. DOI: 10.1016/j.athoracsur.2021.07.026 34389307

[11] Lopez-Delgado JC, Putzu A, Landoni G. The importance of liver function assessment before cardiac surgery: A narrative review. Front Surg. 2022 Dec 6; 9: 1053019. DOI: 10.3389/fsurg.2022.1053019 36561575 PMC9764862

[12] Gundling F, Seidl H, Gansera L, Schuster T, Hoffmann E, Kemkes BM, et al. Early and late outcomes of cardiac operations in patients with cirrhosis: a retrospective survival-rate analysis of 47 patients over 8 years. Eur J Gastroenterol Hepatol. 2010; 22(12): 1466–1473. DOI: 10.1097/MEG.0b013e32834059b6 21346421

[13] Shaheen AA, Kaplan GG, Hubbard JN, Myers RP. Morbidity and mortality following coronary artery bypass graft surgery in patients with cirrhosis: a population-based study. Liver Int. 2009; 29(8): 1141–1151. DOI: 10.1111/j.1478-3231.2009.02058.x 19515218

[14] Steffen RJ, Bakaeen FG, Vargo PR, Kindzelski BA, Johnston DR, Roselli EE, et al. Impact of cirrhosis in patients who underwent surgical aortic valve replacement. Am J Cardiol. 2017; 120(4): 648–654. DOI: 10.1016/j.amjcard.2017.05.034 28693742

[15] Chou AH, Chen TH, Chen CY, Chen SW, Lee CW, Liao CH, et al. Long-term outcomes of cardiac surgery in 1,040 liver cirrhosis patient – nationwide population-based cohort study. Circ J. 2017; 81(4): 476–484. DOI: 10.1253/circj.CJ-16-0849 28163280

[16] Singh V, Savani GT, Mendirichaga R, Jonnalagadda AK, Cohen MG, Palacios IF. Frequency of complications including death from coronary artery bypass grafting in patients with hepatic cirrhosis. Am J Cardiol. 2018; 122(11): 1853–1861. DOI: 10.1016/j.amjcard.2018.08.026 30293650

[17] Xavier S, Norris CM, Ewasiuk A, Kutsogiannis DJ, Bagshaw SM, van Diepen S, et al. The impact of cirrhosis in patients undergoing cardiac surgery: a retrospective observational cohort study. Can J Anaesth. 2020; 67(1): 22–31. DOI: 10.1007/s12630-019-01493-7 31571117

[18] Macaron C, Hanouneh IA, Suman A, Lopez R, Johnston D, Carey WW. Safety of cardiac surgery for patients with cirrhosis and Child-Pugh scores less than 8. Clin Gastroenterol Hepatol. 2012; 10(5): 535–539. DOI: 10.1016/j.cgh.2011.12.030 22210437

[19] Ruiz-Morales J, Ivanova-Georgieva R, Fernández-Hidalgo N, García-Cabrera E, Miró JM, Muñoz P, et al; Spanish Collaboration on Endocarditis Group-Grupo de Apoyo al Manejo de la Endocarditis en España (GAMES); Spanish Network for Research in Infectious Diseases (REIPI). Left-sided infective endocarditis in patients with liver cirrhosis. J Infect. 2015; 71(6): 627–641. DOI: 10.1016/j.jinf.2015.09.005 26408206

[20] Moher D, Liberati A, Tetzlaff J, Altman DG; PRISMA Group. Preferred reporting items for systematic reviews and meta-analyses: the PRISMA statement. PLoS Med. 2009; 6(7): e1000097. DOI: 10.1371/journal.pmed.1000097 19621072 PMC2707599

[21] Stang A. Critical evaluation of the Newcastle-Ottawa scale for the assessment of the quality of nonrandomized studies in meta-analyses. Eur J Epidemiol. 2010; 25(9): 603–605. DOI: 10.1007/s10654-010-9491-z 20652370

[22] Ioannidis JP. Interpretation of tests of heterogeneity and bias in meta-analysis. J Eval Clin Pract. 2008; 14(5): 951–957. DOI: 10.1111/j.1365-2753.2008.00986.x 19018930

[23] Higgins JP, Thompson SG, Deeks JJ, Altman DG. Measuring inconsistency in meta-analyses. BMJ. 2003; 327(7414): 557–560. DOI: 10.1136/bmj.327.7414.557 12958120 PMC192859

[24] Filsoufi F, Rahmanian PB, Castillo JG, Chikwe J, Kini AS, Adams DH. Results and predictors of early and late outcome of coronary artery bypass grafting in patients with severely depressed left ventricular function. Ann Thorac Surg. 2007; 84(3): 808–816. DOI: 10.1016/j.athoracsur.2007.04.117 17720380

[25] Hayashida N, Shoujima T, Teshima H, Yokokura Y, Takagi K, Tomoeda H, et al. Clinical outcome after cardiac operations in patients with cirrhosis. Ann Thorac Surg. 2004; 77(2): 500–505. DOI: 10.1016/j.athoracsur.2003.06.021 14759426

[26] An Y, Xiao YB, Zhong QJ. Open-heart surgery in patients with liver cirrhosis. Eur J Cardiothorac Surg. 2007; 31(6): 1094–1098. DOI: 10.1016/j.ejcts.2007.01.042 17314050

[27] Suman A, Barnes DS, Zein NN, Levinthal GN, Connor JT, Carey WD. Predicting outcome after cardiac surgery in patients with cirrhosis: a comparison of Child-Pugh and MELD scores. Clin Gastroenterol Hepatol. 2004; 2(8): 719–723. DOI: 10.1016/S1542-3565(04)00296-4 15290666

[28] Teh SH, Nagorney DM, Stevens SR, Offord KP, Therneau TM, Plevak DJ, et al. Risk factors for mortality after surgery in patients with cirrhosis. Gastroenterology. 2007; 132(4): 1261–1269. DOI: 10.1053/j.gastro.2007.01.040 17408652

[29] Morisaki A, Hosono M, Sasaki Y, Kubo S, Hirai H, Suehiro S, et al. Risk factor analysis in patients with liver cirrhosis undergoing cardiovascular operations. Ann Thorac Surg. 2010; 89(3): 811–817. DOI: 10.1016/j.athoracsur.2009.12.021 20172135

[30] Arif R, Seppelt P, Schwill S, Kojic D, Ghodsizad A, Ruhparwar A, et al. Predictive risk factors for patients with cirrhosis undergoing heart surgery. Ann Thorac Surg. 2012 Dec; 94(6): 1947–52. DOI: 10.1016/j.athoracsur.2012.06.057 22921237

